# Plate and K-Wire Show Advantages to Nailing for Distal Diametaphyseal Radius Fracture in Children: A Retrospective, Two-Center Study

**DOI:** 10.3390/jcm14134626

**Published:** 2025-06-30

**Authors:** Frederik Weil, Lucas Fabarius, Luisa Weil, Paul A. Grützner, Michael Boettcher, Christel Weiß, Stefan Studier-Fischer

**Affiliations:** 1 Department of Trauma and Orthopaedic Surgery, BG Trauma Center Ludwigshafen, Heidelberg University Hospital, 67071 Ludwigshafen, Germany; 2 Department of Pediatric Surgery, University Medical Center Mannheim, University Heidelberg, 68167 Mannheim, Germany; 3 Department of Medical Statistics, Biomathematics and Information Processing, University Medical Center Mannheim, University Heidelberg, 68167 Mannheim, Germany

**Keywords:** children, fracture, diametaphysis, plate osteosynthesis, K-wire, ESIN, TEN, complications

## Abstract

**Background/Objectives**: Distal forearm fractures are the most common fractures in children. Three surgical techniques are most commonly used at the level of the radial diametaphysis on the distal forearm in children: K-wire, ascending ESIN (elastic stable intramedullary nail) or plate osteosynthesis. The aim of this study was to compare these procedures in children with distal diametaphyseal radius fractures regarding operative and functional outcome. **Methods**: A retrospective study was conducted in two level 1 trauma centers. Children and adolescents aged 2 to 15 years were included. The study period was from January 2010 to December 2022. The hospital information system was used to record patient age, gender, height, weight, fracture location, degree of angular deformity postoperatively, surgical procedure and postoperative complications, which were described in the medical records of the hospital information system. Complications graded by modified Clavien–Dindo–Sink served as the primary outcome. Reduction accuracy, operative and fluoroscopy times, immobilization length and postoperative motion were the secondary endpoints. **Results**: A total of 213 children were included in the study. K-wire osteosynthesis was performed in 25%, nailing in 19% and volar plate osteosynthesis in 55%. All ESIN were inserted in ascending technique. Complications occurred in 22% of patients and did not differ overall between techniques (*p* = 0.20). Severe complications were significantly more frequent after ESIN (20%) than after K-wires (7%) or plates (4%) (*p* = 0.04). Plate fixation achieved the most accurate alignment (≤5° angular deformity in 93% vs. 57% K-wires and 61% ESIN; *p* < 0.0001) and the fewest late motion restrictions (*p* = 0.02). K-wire surgery was fastest technique and required the least fluoroscopy, but necessitated the longest postoperative cast. **Conclusions**: Volar plating combines reliable anatomical reduction with a low rate of major complications and early mobilization, supporting its use in older children whose remodeling potential is limited. K-wires are a swift, minimally invasive option for younger patients, albeit with less precise reduction and prolonged immobilization. Conventional ESIN showed the highest burden of severe complications.

## 1. Introduction

Forearm fractures are the most common fractures in childhood. The distal third of the forearm is most commonly affected, accounting for almost 75% of all forearm fractures [1,2]. The growth plate of the distal radius accounts for 80% of the longitudinal growth of the forearm [3], so there is a remarkable potential for remodeling, particularly in younger children, and even gross deformities can grow straight during the course of longitudinal growth [4]. However, the remodeling potential depends not only on the patient’s age, but also on the distance of the fracture from the distal joint. The closer the fracture is to the distal growth plate, the greater the potential for remodeling. A fracture in the shaft area of one of the two forearm bones should always be considered a joint fracture, as the structure of the middle radioulnar joint, which is formed by the ulna, the radius and the interosseous membrane, is disturbed by a fracture-related angular deformity. If the fracture is located in the shaft area, the forearm rotation, in which the radius rotates around the ulna, is disturbed if the angular deformity causes a blockage during the forearm rotation. Even if the angular deformity remodels to a straight axis after several years, a disturbance of the forearm rotation remains, as the interosseous membrane and the proximal and distal radioulnar joint have adapted to the limited forearm rotation [5]. The time remaining for remodeling to a straight axis and restoring full mobility of the pronation and supination is therefore limited. If the remaining, age-dependent correction potential and the time remaining for remodeling the axis are not sufficient, the limited forearm rotation will remain for life. A larger angular deformity in the diaphyseal area must therefore not be tolerated even in smaller children [6]. The remodeling potential for metaphyseal fractures, on the other hand, is enormous [4,7]. The remodeling potential at the diametaphyseal junction is not known.

K-wire osteosynthesis has become established for the surgical treatment of metaphyseal fractures of the distal radius in children [8]. For diaphyseal fractures, the osteosynthesis of choice is nailing [9]. Due to the technical difficulties with ESIN (elastic stable intramedullary nail) osteosynthesis in the area of the diametaphyseal junction on the distal radius, modifications of nailing have been described in recent years in order to achieve a good reduction in the fracture using minimally invasive nailing. Krohn et al. described the double buckling technique [10]. Furthermore, the entry point of the ESIN osteosynthesis was changed and the nail was inserted antegrade instead of retrograde via a minimally invasive Thompsen approach [11]. To date, however, no gold standard has been developed, as each osteosynthesis procedure has its own specific disadvantages, some of which can lead to long-term functional limitations or revision surgery.

K-wire and nailing are minimally invasive procedures. Small surgical incisions remote from the fracture are sufficient to insert the material, whereas larger access points are required for plate osteosynthesis. The stability provided after nailing and plate osteosynthesis allows for early functional exercise of the wrist, whereas postoperative immobilization of the wrist occurs after K-wire osteosynthesis. One disadvantage of plate osteosynthesis is the need for a second operation to remove the osteosynthesis material. This disadvantage is also evident in nailing. In contrast, the removal of wires after K-wire osteosynthesis does not require further anesthesia if the K-wires are left epicutaneous.

Plate osteosynthesis has no longer played a major role in the surgical treatment of children, at least since the development of ESIN [12]. In contrast, plate osteosynthesis represents the majority of osteosynthesis procedures in the treatment of distal radial fractures in adults. Volar plate osteosynthesis is the most common form of osteosynthesis, particularly for distal radius fractures in adults [13]. One of the specific disadvantages of plate osteosynthesis is that it is highly invasive, which contradicts child-friendly, gentle treatment. Nevertheless, plate osteosynthesis also appears to have few major complications in children and it appears to be a safe, albeit invasive, osteosynthesis procedure [14].

This study aimed to compare complications, surgical and radiation exposure time, reduction quality and postoperative function in common three osteosynthesis techniques at two Level I trauma centers for diametaphyseal fractures of the distal radius at growth age.

## 2. Materials and Methods

### 2.1. Software

**Figure 1:**
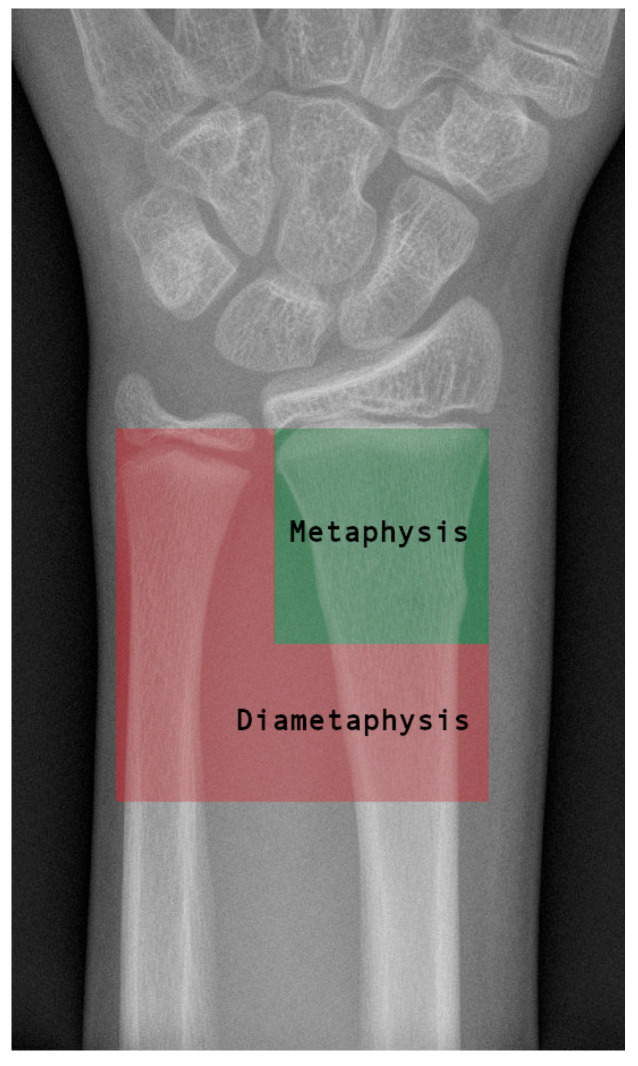
Definition of the diametaphysis: A green square was placed over the joint of the radius and a red square over the joint of the radius and the ulna. The diametaphysis of the radius is defined as the area within the red square that lies proximal to the green square. The diametaphysis refers exclusively to the area of the radius and not to the ulna.

**Figure 2:**
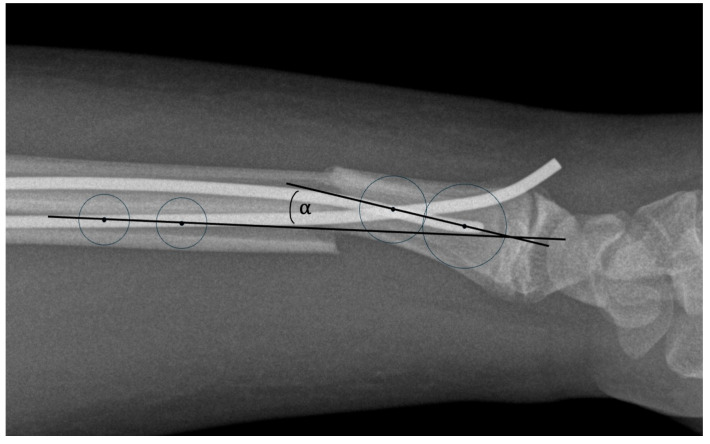
Measurement of the angular deformity of the shaft in sagittal plane after fracture. The angle alpha describes the size of the angular deformity in the sagittal plane.

**Figure 3:**
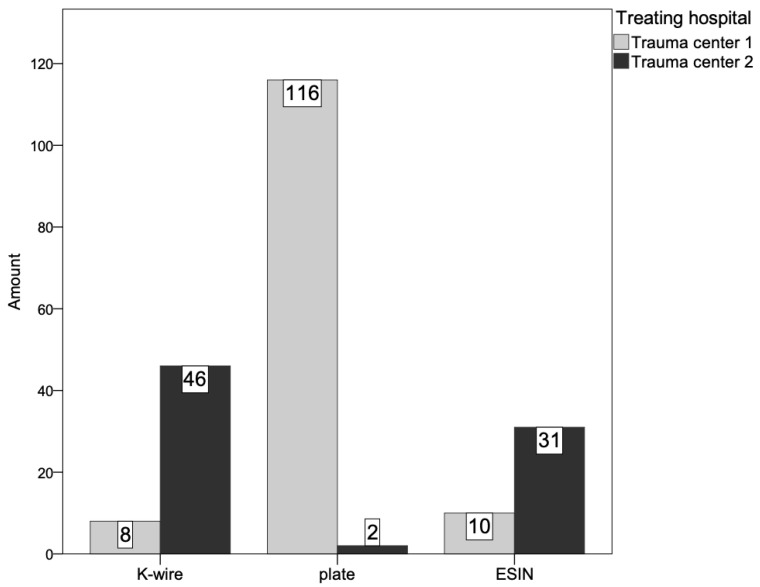
Distribution of the different forms of osteosynthesis between the two trauma centers.

**Figure 4:**
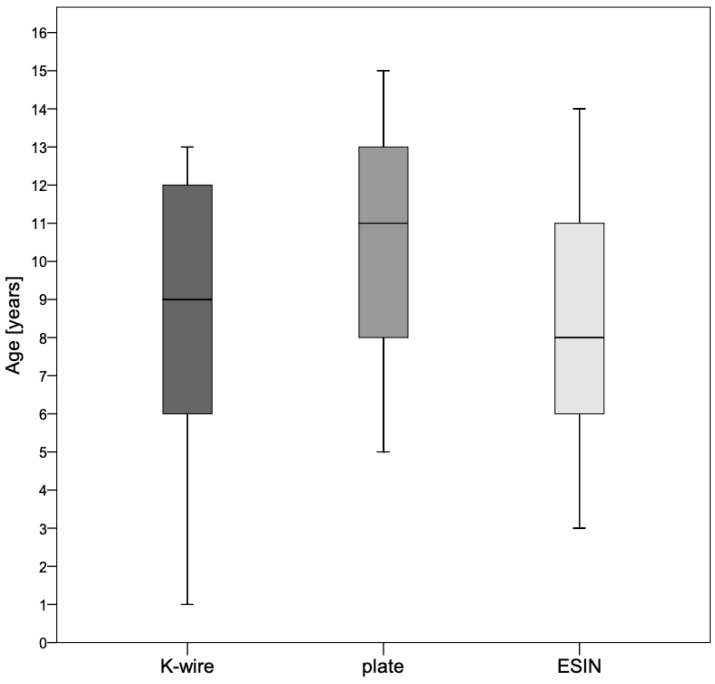
Age distribution by osteosynthesis. Older patient age (*p* < 0.0001) was significantly more frequently associated with plate osteosynthesis. However, there were no differences in age between the K-wire- and the ESIN-group.

**Figure 5:**
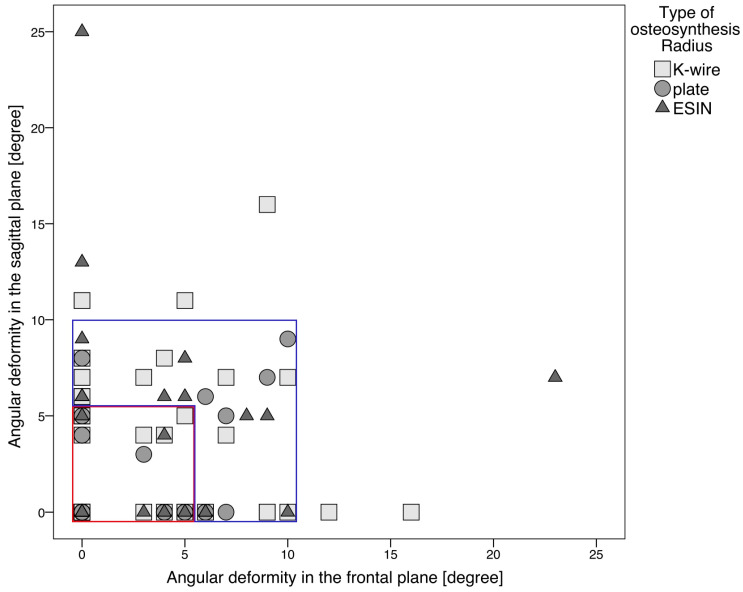
Reduction quality depending on the osteosynthesis procedure. Each symbol (triangle, square or circle) represents one patient. The measurement of the angular deformity in the frontal and sagittal plane was recorded in the postoperative X-ray. The symbols within the red square belong to group 1. An angular deformity of less than or equal to 5 degrees was measured in these patients in the sagittal or frontal planes. The symbols within the blue square belong to group 2. Here, an angular deformity of 6 to 10 degrees was measured postoperatively in the sagittal or frontal plane. All other patients belong to group 3. In these patients, an angular deformity greater than 10 degrees was measured in the sagittal or frontal plane. There were no patients in group 3 who had undergone plate osteosynthesis. A postoperative restriction of movement was significantly more frequently associated with a poorer reduction result (*p* = 0.02).

**Figure 6:**
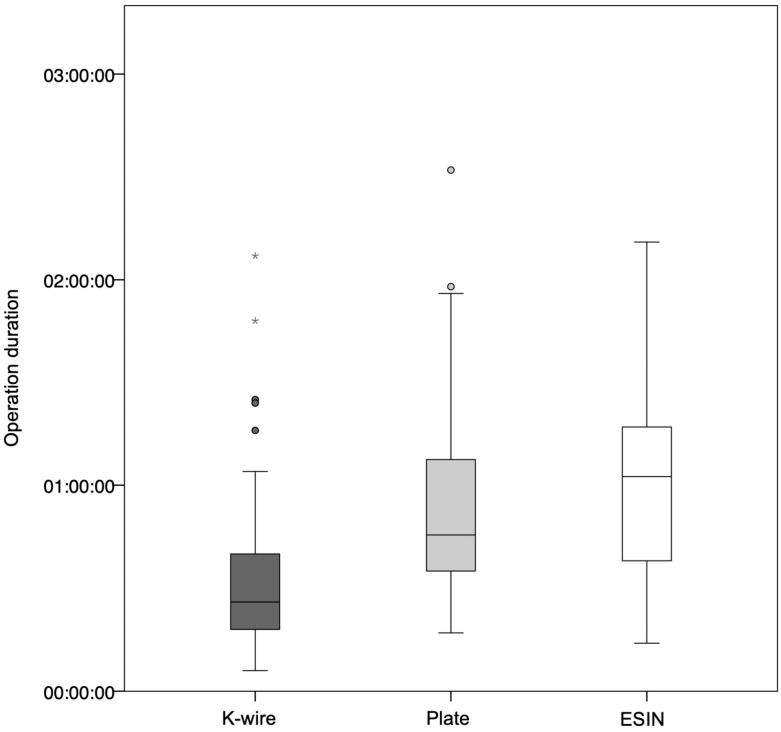
Operation duration depending on the osteosynthesis procedure. The operation duration for K-wire osteosynthesis was significantly shorter than for nailing and plate osteosynthesis (*p* < 0.0001). In contrast, there was no significant difference in operation duration between plate osteosynthesis and nailing (*p* = 0.07). Outliers in the duration of surgery that were more than three times the interquartile range were marked with an asterisk.

The necessary patient data was collected via the respective clinic program (SAP i.s.h.med^®^, SAP Deutschland SE & Co. KG, Walldorf, Germany; Medico^®^, CompuGroup Medical SE & Co. KGaA, Koblenz, Germany). The fracture localization, fracture mechanism and pre- and postoperative angular deformity were measured and evaluated using digital X-ray images and the PAC system (Syngo (Simens Healthineers^®^, Erlangen, Germany) or IMPAX (agfa health care^®^, Mortsel, Belgium)) of the respective clinic. Figures 1 and 2 were created using GIMP (GNU Image Manipulation Program) 3.0.2. The statistical analysis was carried out with the support of Prof. Dr. Christel Weiß, Head of the Department of Medical Statistics, Biomathematics and Information Processing at the Mannheim Medical Faculty using the statistical program package SAS Analytic Software, Release 9.4 (SAS Institute Inc., Cary, NC, USA). Figures 3–6 were created using SPSS, Release 24.0 (IBM, New York, NY, USA). The AI-powered translation solution DeepL Translator (DeepL SE, Cologne, Germany) was used as a translation aid. No other artificial intelligence program was used.

### 2.2. Data Collection

A retrospective study on the surgical treatment of diametaphyseal distal radius fractures in children and adolescents was conducted in two level 1 trauma centers. Children and adolescents aged 2 to 15 years were included. The study period was from January 2010 to December 2022. The exclusion criteria were fractures at the level of the epiphysis, metaphysis and diaphysis, as well as pathological fractures. The diametaphyseal junction was defined as the area between the square under the distal radius joint and the square under the distal radius and ulna joint taken together [12]. Figure 1 shows the area of the diametaphysis.

One of the two level I trauma centers exclusively employs specialists in trauma surgery. In this trauma center, plate osteosynthesis was predominantly used, but K-wire osteosynthesis and nailing were also used at the level of the diametaphyseal junction of the distal radius in children. The other level I trauma center is staffed exclusively by specialists in pediatric surgery. Fractures at the level of the diametaphyseal junction of the distal radius are regularly treated using K-wire or ESIN osteosynthesis in this trauma center, and plate osteosynthesis was performed in only two cases. Every operation was performed either by a specialist in pediatric surgery or by a specialist in trauma surgery. Several surgeons were involved in both the K-wire and plate osteosynthesis and in the nailing procedures.

### 2.3. Clinical Examination and Functional Outcome

One of the two trauma centers provided structured follow-up care with several check-ups, including implant removal. At the second trauma center, follow-up examinations were transferred to the outpatient practice, with close contact between outpatient practices and the trauma center, and any complications were reported immediately. Implant removal for the center’s own patient group was generally also carried out at the second trauma center, with clinical follow-up taking place at this stage at the latest. Follow-up examinations were carried out in both trauma centers by means of clinical follow-up with measurement of range of motion and describing the scar, as well as documentation of any abnormalities (abnormalities in the scar, supination, pronation, wrist extension, and flexion). X-rays were also taken when indicated. The follow-up examinations were carried out in both trauma centers by different residents and specialists, who may not have been present during the operation. The complications described (except for intraoperative procedural changes) were identified by the follow-up examinations, accordingly.

### 2.4. Complications

The main outcome measure was postoperative complications depending on the surgical procedure. The complications were classified using a modified form of the Clavien–Dindo–Sink classification. In their study, Dodwell et al. found “good inter-rater and excellent intrarater reliability for the evaluation of complications following pediatric orthopedic upper extremity” [15] using the modified form of the Clavien–Dindo–Sink classification [15]. Subsequently, a distinction was made between mild (modified Clavien–Dindo–Sink type 1 and 2) and severe (modified Clavien–Dindo–Sink type 3 and 4) complications.

Referring to the movement restrictions, there was neither a differentiation in the severity of the restrictions’ grade, nor a distinction between restriction of pronation/supination or wrist extension/flexion. The extent of the restriction was not further subdivided. Only the duration of the restriction was subdivided. A described restriction of movement more than 3 months after surgery was classified as severity grade 1, a described restriction of movement 3 to 6 months after surgery was classified as severity grade 2, and a described restriction of movement beyond 6 months was classified as severity grade 3 according to the modified Clavien–Dindo–Sink classification. In accordance with our subdivision into mild (modified Clavien–Dindo–Sink 1 and 2) and severe (modified Clavien–Dindo–Sink 3 and 4) complications, movement restrictions up to 6 months were classified as mild complications and movement restrictions lasting longer than 6 months were classified as severe complications.

Secondary outcome measures were procedure-dependent complications in relation to patient age, height and weight, operation duration, reduction quality, fracture mechanism and fracture type. In addition, the radiation exposure time and the dose area product from intraoperative X-ray imaging were compared depending on the osteosynthesis procedure.

### 2.5. Radiological Measurement of the Reduction Quality

In a further analysis, the reduction quality was examined as a function of the osteosynthesis procedure. The quality was divided into three groups, whereby the classification was based on the angular deformity in the sagittal and frontal plane measured in the intraoperative or first postoperative X-ray. The first postoperative X-ray was performed on the 1st or 2nd postoperative day. The measurements of angular deformity were performed by a resident surgeon who was not present during any of the operations performed. Supervision was provided by a specialist for trauma surgery, who was the operating surgeon in very few of the osteosynthesis procedures performed. Inter-rater reliability was not assessed.

The angular deformity was measured as the following: in the proximal and distal shaft fragments relative to the fracture, two circles were drawn tangentially to the two cortices of the shaft and a straight line was drawn through the centers of the tow circles. The angle between the two straight lines was measured. Figure 2 shows an example of this.

Based on the paper by Stark et al., the classification criteria were good reduction results in postoperative angular deformity measured in the sagittal or frontal plane ≤ 5 degrees, moderate results in 6–10 degrees and poor reduction results in > 10 degrees [16].

### 2.6. Statistical Analysis

The anonymized data were first inserted into a cumulative Excel spreadsheet, then imported into SAS and analyzed descriptively. The mean, median and standard deviation were calculated for quantitative variables. Absolute and relative frequencies are given for qualitative factors. Median, absolute and relative frequencies were calculated for normally distributed variables.

The comparison of different osteosynthesis procedures with regard to the incidence of complications focused on distal diametaphyseal radius. A one-factorial analysis of variance was performed to compare three groups on a normally distributed variable (specifically age, height and weight). If the results were significant, Scheffé post hoc tests were performed. The comparison of non-normally distributed variables of the operative and postoperative parameters between the different osteosynthesis procedures was performed using a Kruskal–Wallis test. The duration of surgery, length of hospital stay, radiation exposure time and dose area product were analyzed. First, the demographic parameters of the different treatment groups of the respective fracture height were compared using ANOVA.

The Pearson chi-square test was used to compare categorical variables, in particular between treatment groups, complication occurrence and the reduction result. In cases where the requirements for the chi-square test were not met, a Fisher exact test was used. A two-sample t-test, a Wilcoxon rank sum test, a chi-square test or a Fisher exact test were used to compare two groups. The comparison of the operative and postoperative parameters between the different osteosynthesis procedures was carried out using the Wilcoxon rank sum test. The duration of surgery, length of hospital stay, radiation exposure time and dose area product were analyzed.

The influence of the reduction result on the occurrence of postoperative movement restrictions (transient or permanent) was investigated using the Cochran–Armitage trend test.

The comparison of the operative and postoperative parameters between the different osteosynthesis procedures was carried out using the Wilcoxon rank sum test. The duration of surgery, length of hospital stay, radiation exposure time and dose area product were analyzed.

The pooled T-test was used to analyze the influence of patient age and anthropometry on the occurrence of complications. The significance level was set at a *p*-value < 0.05 in order to identify statistically significant differences and correlations α = 0.05.

## 3. Results

### 3.1. Data Collection

Of the 217 patients, 213 children were included in the study. Four patients were excluded due to complications that were not attributable to osteosynthesis of the radius. K-wire osteosynthesis was performed in 25%, nailing in 19% and volar plate osteosynthesis in 55%. Of the 41 ESIN osteosyntheses, 39% were inserted radially, just proximal to the growth plate at the level of the styloid process and 61% via the lister tubercle. All ESIN were inserted in ascending technique. The distribution of the different forms of osteosynthesis between the two trauma centers is shown in Figure 3. The fracture healed without delay in all patients. No pseudarthrosis was observed.

The gender distribution of the groups was without significant differences. A total of 63% of the patients were treated at one trauma center, and 37% at the other. Older patient age (*p* < 0.0001), taller height (*p* = 0.01) and higher patient weight (*p* < 0.0001) were significantly more frequently associated with plate osteosynthesis, whereas there was no significant difference between K-wire and nailing. The anthropometric data depending on the osteosynthesis procedure are described in Table 1.

The age distribution by osteosynthesis is shown in Figure 4.

### 3.2. Complications and Influencing Factors

Across all groups, 47 complications occurred with a proportion of 22%. Hypertrophic scarring was particularly common after plate osteosynthesis. Functional impairment due to hypertrophic scarring was never documented. In three cases, there was a postoperative infection of the surgical wound. In two cases, this infection occurred after K-wire osteosynthesis, both of which were treated with antibiotics. In one case, there was a superficial infection at the ESIN insertion site. This was treated with local antiseptic creams without antibiotics and healed without complications. In one case in the K-wire group, a broken K-wire was found within the epiphysis, which was left in place as it was located outside the joint. In another case involving the K-wire group, postoperative sensory disturbances were observed in the superficial branch of the radial nerve. These completely regressed after a few months. In two cases in the ESIN group, the postoperative X-ray image was assessed as insufficiently well-reduced, resulting in early revision surgery. A total of two iatrogenic tendon injuries occurred. One was caused by a K-wire that ran through the tendon of the extensor carpi radialis brevis tendon, and revision surgery was performed promptly due to functional complaints. Another time, a lesion of the extensor pollicis longus tendon was observed due to the ESIN being inserted over the tuberculum listeri. Revision surgery was also performed in this case. In one case, following plate osteosynthesis, a peri-implant fracture occurred after the patient fell again with the plate osteosynthesis still in place. In another case following plate osteosynthesis, removal of the plate and minor trauma resulted in a fracture through a former screw hole. In three other cases, refracture was observed in the former fracture site after removal of the plate osteosynthesis. The plate was removed twice three months after the fracture and once four months after the fracture. In four cases, the osteosynthesis material migrated, which led to revision surgery in each case. Twice, the K-wire migrated under the skin, and twice, the radial ESIN migrated distally, resulting in skin irritation and revision surgery. During nailing, there were intraoperatively two unplanned changes in the surgery procedure. In one case, a fracture occurred during ESIN insertion, causing the ESIN to lose its hold in the bone and requiring a different procedure to be selected. In another case, intraoperative nailing did not result in a satisfactory reduction, so a different osteosynthesis procedure was also selected here. Table 2 shows the description of the complications.

In the statistical analysis, no procedure proved to be superior with regard to the occurrence of complications (*p* = 0.20). In patients with K-wire 9/54 (17%), in ESIN, 13/41 (32%) and in plate, 25/118 (21%) had a complication. The occurrence of complications and the severity classification are shown in Table 3. No Clavien 4 complications were observed across all groups.

In a statistical sub-analysis, only the complications were compared between all three groups and this showed a significantly higher incidence of severe complications in the nailing group vs. the K-wire and plate group (*p* = 0.04). The number of K-wires showed no significant difference in the incidence of complications (*p* = 0.25). The entry of the ESIN at the radius had no significant influence on the incidence of complications (*p* = 0.66).

The occurrence of complications was independent of gender (*p* = 0.62). The fracture mechanism (extension or flexion) had no influence on the occurrence of complications (*p* = 0.13). The fracture type had a significant influence on the occurrence of complications. Complications occurred significantly more frequently in complete fractures than in greenstick fractures (*p* = 0.04). However, the severity of complications was not dependent on the fracture type (*p* = 0.1).

Age (*p* = 0.22), height (*p* = 0.77), weight (*p* = 0.07) and BMI (*p* = 0.47) had no influence on the occurrence of complications. The latency between the diagnosis of the fracture and the start of surgery was independent of the occurrence of complications (*p* = 0.88).

A total of 6.6% of all fractures were open fractures. The soft tissue damage or whether an open or closed fracture was present had no influence on the occurrence of complications (*p* = 0.52). In 4.7% of cases, the distal diametaphyseal radius fracture was accompanied by multiple injuries. One patient suffered polytrauma.

### 3.3. Reduction Quality and Its Functional Outcome

In addition to the occurrence of complications, the quality of reduction was also examined depending on the osteosynthesis method. There was a significant difference in the reduction result with greater angular deformity with K-wire or nailing compared to plate osteosynthesis (*p* < 0.0001). Figure 5 and Table 4 show the deviations measured after reduction depending on the osteosynthesis procedure.

In one case, the restriction of wrist movement was related to the extension and flexion, with a clearly protruding ESIN end. The axis measured postoperatively was straight in this case. The restriction of movement was completely reversed after implant removal or after 6 months. In four other cases, the restriction of wrist movement was related to rotational movement. The postoperative axis measurement showed a clear angular deformity in each case. In two cases, the restriction of movement persisted for more than six months, and in two cases it decreased after six months.

A postoperative restriction of movement was significantly more frequently associated with a poorer reduction result (*p* = 0.02).

### 3.4. Hospital Stay, Radiation Exposure Time and Postoperative Immobilization

After K-wire osteosynthesis, both centers had a similar follow-up treatment plan. At both centers, immobilization was achieved with a cast for 3–6 weeks, with an upper arm or forearm cast applied depending on the extent of ulnar involvement. The duration of immobilization was determined by the patient’s age, with older patients undergoing a longer immobilization phase. The follow-up treatment regimen after nailing and plate osteosynthesis varied greatly and was always determined by the surgeon based on the estimated stability of the osteosynthesis in relation to the fracture. If the ulna was affected without osteosynthesis, the arm was regularly immobilized in a cast. After K-wire osteosynthesis, the duration of immobilization in the forearm cast was significantly longer (*p* < 0.001).

The duration of surgery for K-wire osteosynthesis was significantly lower than that for plate osteosynthesis or nailing (*p* < 0.001). There was no significant difference in the duration of surgery between plate osteosynthesis and nailing (*p* = 0.07). Figure 6 illustrates the duration of surgery depending on the osteosynthesis procedure. A longer operation time was significantly more frequently associated with complications (*p* = 0.02).

The radiation exposure time was significantly shorter with K-wire osteosynthesis than with plate osteosynthesis (*p* = 0.03) and shorter than with nailing (*p* = 0.04), whereas there was no significant difference in radiation exposure time between nailing and plate osteosynthesis (*p* = 0.6).

## 4. Discussion

### 4.1. Complications and Influencing Factors

In this retrospective two-center study, three established techniques (K-wire, ESIN and volar plate) for pediatric diametaphyseal distal radius fractures were compared. Although the overall complication rate did not differ significantly between techniques, severe (Clavien 3) complications were significantly more common after nailing than after K-wires or plates. None of the surgery procedures resulted in particularly serious complications (Clavien 4).

The high complication rate of nailing compared to plate and K-wire is especially striking. In nailing, the major complications were persisting angular deformity due to insufficient reduction, so the decision to revise was made twice postoperatively and once intraoperatively. On one occasion, there was an intraoperative fracture of the distal fracture fragment along the dorsal corticalis, which serves as a support for the ESIN, so that with a consecutive lack of stability due to the ESIN, a reduction in alignment could not be achieved and the osteosynthesis procedure had to be changed intraoperatively. The entry point of the ESIN had no influence on the occurrence of complications. Overall, complications occurred in 22% of patients, with no significant difference in the overall complication rate between the procedures.

While nailing remains the gold standard for mid-shaft pediatric forearm fractures [9], the technique is technically demanding at the distal diametaphyseal junction. Our sub-analysis showed a significantly higher rate of severe complications with nailing, including iatrogenic tendon injury and angular deformity requiring revision. Recent antegrade or double-bending modifications [10,11] were not used in the present series. By contrast, experienced trauma surgeons routinely performing adult volar plating achieved consistently low complication rates, suggesting that surgeon familiarity is a critical determinant of outcomes.

However, there are some concerns that necessitate a review of routine use of plate osteosynthesis at the diametaphyseal junction. The major complications of plate osteosynthesis are striking. One case showed an implant-associated fracture with fracture after minor trauma in the area of a former screw bearing, and three cases showed a refracture in the former fracture area. In the three cases of refracture, the plate osteosynthesis was removed twice after 3 months and once after 4 months. The refractures are most likely due to premature implant removal. Kubiak et al. recommend treating diametaphyseal fractures like diaphyseal fractures with regard to the refracture rate [17], so that the recommendation should be to remove the plate after 6 months at the earliest [18,19].

In plate osteosynthesis, the occurrence of a hypertrophic scarring was the main complication observed. Even if the complication of a hypertrophic scar is classified as mild, it can still lead to esthetic impairments, functional limitations, emotional stress, and psychological damage, which can place a considerable burden on patients [20]. In our patient group, no functional deficit was reported as a result of the hypertrophic scarring following plate osteosynthesis. Nevertheless, the affected patients and their parents complained about the unsightly appearance.

Interestingly, neither patient weight nor height had any influence on the occurrence of complications. Prior to the study, it was assumed that a higher patient weight could potentially lead to a higher complication rate [21]. However, no significant correlation between patient weight, age, height and the occurrence of complications could be observed.

The statistical analysis also showed that a longer operation duration was significantly more frequently associated with a higher risk of complications. This is consistent with the generally known hypothesis that a longer surgery duration leads to an increased incidence of complications [22].

Among the serious complications, it should also be noted that, compared to plate osteosynthesis, K-wire osteosynthesis and nailing resulted in fewer cases of migration of the osteosynthesis material, which necessitated revision surgery. This suggests that plate osteosynthesis provides greater stability within the bone and consecutively bridges the fracture to a greater extent than the other two osteosynthesis procedures mentioned. It should be added that no secondary dislocation was observed after any of the osteosynthesis procedures.

After K-wire osteosynthesis, a complication occurred in 17% of cases, after nailing in 32% of cases and after plate osteosynthesis in 22% of cases. This largely corresponds to the data in the literature and underlines the difficulty of the diametaphyseal junction zone [17,23,24,25].

### 4.2. Hospital Stay, Radiation Exposure Time and Postoperative Immobilization

It was shown that K-wire osteosynthesis was associated with the shortest operation time and the lowest radiation exposure. However, it would have been expected that the radiation exposure would be lowest with open reduction in the context of plate osteosynthesis. Plate osteosynthesis was mainly performed by trauma surgeons, whereas K-wire osteosynthesis was performed by pediatric surgeons. It is conceivable that pediatric surgeons attach greater importance to lower radiation exposure than trauma surgeons.

No standardized procedure was observed with regard to postoperative immobilization. Follow-up treatment varied between the two trauma centers, within a single trauma center, and between different surgeons. However, both trauma centers showed significantly longer postoperative immobilization after K-wire osteosynthesis. The advantage of greater stability of nailing and plate osteosynthesis more often led to early functional exercise and consequently less postoperative restriction for patients.

Since plate osteosynthesis is the most invasive of the three osteosynthesis procedures, it was assumed that the duration of inpatient treatment would be longest for plate osteosynthesis. This was not found to be the case. There was no significant difference in the length of hospitalization.

### 4.3. Reduction Quality and Its Functional Outcome

A longer-lasting restriction of movement after surgery correlated with greater angular deformity in terms of a poorer reduction result. A change in the alignment disturbs the rotation of the forearm both diaphyseally and metaphyseally [26]. Plate osteosynthesis showed significantly better reduction results compared to the other procedures. While 93% of cases were rated as “good” for plate osteosynthesis, this percentage was significantly lower for K-wire and ESIN osteosynthesis. In addition to Colaris et al., the present study also showed that an angular-correct reduction result must be enforced intraoperatively in order to avoid a postoperative limited forearm rotation [26]. The agreement regarding the importance of exact reduction should therefore be particularly emphasized: Khan et al. point out that rotational malalignments in particular do not remodel reliably and should therefore be corrected surgically [27]. This assessment is supported by the available data, according to which poorer reduction results were significantly more frequently associated with movement restrictions.

Regarding the quality of initial reduction, plate fixation almost eliminated reductions with > 10° axis deviation, whereas nailing and K-wires left a moderate or poor axis in 39% and 43% of cases, respectively. Given that remodeling potential diminishes with increasing distance from the distal physis and patient age, the stability and anatomic alignment provided by plates are particularly attractive for older children. The trade-offs are a larger incision, a longer operative time and the need for a second narcosis (implant removal). These findings invite a paradigm shift: for older children and adolescents with diametaphyseal distal radius fractures that have limited remodeling potential, primary volar plating should be strongly considered. K-wires remain appropriate for younger children with substantial remodeling potential, whereas ESIN may be reserved for mid-shaft patterns.

### 4.4. Strengths and Limitations

Although the results presented here are very relevant for pediatric trauma clinics when considering surgical strategy, this study has certain limitations. Strengths include (i) the largest comparative cohort to date, (ii) treatment in two high-volume level-I centers with established, technique-specific expertise, reducing learning-curve bias, and (iii) systematic complication grading with modified Clavien–Dindo–Sink.

Limitations are those inherent to retrospective designs. They include selection bias (surgeons chose the implant); the lack of structured, targeted follow-up care for all patients who have undergone surgery; depending on the examiner, differently well-documented examination of the operated patient; incomplete capture of mild complications treated outside the hospital; and absence of patient-reported outcome measures.

Furthermore, there are also methodological limitations: follow-up ended after implant removal, so late growth disturbances—while unlikely given the plate position— or improvement in the range of motion of the wrist cannot be excluded entirely. The measurement of angular deformity was performed by only one person, so despite supervision, it is possible that errors were made. The measurement of the range of motion was carried out by different people; therefore, differences in measurement techniques and results cannot be ruled out. In addition, the age range of the patients examined was very wide, from 2 to 15 years. It is possible that the 2-year-old child was examined differently than the 15-year-old, so that errors may have been made in determining the range of motion. Another limitation of the study is that two different disciplines and many different surgeons were involved in the surgical treatment of the injured children. The choice of osteosynthesis could be based on the specific center and individual surgeon preferences. A different approach to follow-up examinations and documentation can be assumed. This creates a strong confounding variable. This expertise bias likely impacted complication rates and outcomes.

## 5. Conclusions

Plate fixation yielded markedly better reduction than either minimally invasive (K-wire and ESIN) method, and this translated into fewer long-term motion restrictions. K-wires required the shortest operative time and the least intra-operative radiation exposure, but necessitated prolonged postoperative casting. Plates and nailing allowed early mobilization, yet nailing carried the longest fluoroscopy times and the highest proportion of re-operations. In most cases of K-wires fixation, a second anesthesia was not necessary.

Finally, the results of this retrospective cohort study suggest that, especially in older children or adolescents with limited remodeling potential, a proper axis reduction in diametaphyseal distal radius fractures should be aimed for in order to safely prevent functional impairment. In the present study, plate osteosynthesis and K-wires showed the lowest complication rates. K-wires are minimally invasive, and are easy and fast to insert. They are associated with the lowest radiation exposure time, and there is no need for a second narcosis. However, in older children with a low potential for remodeling, a plate osteosynthesis may be chosen, with excellent results and minimal risk of serious complications. The next step should be a prospective study in which the 213 patients who underwent surgery are followed up. This will allow the collection of patient-reported outcome measures and measurement of the range of motion of the wrist several years after surgery in order to eliminate the methodological weaknesses of this retrospective study.

Future studies should compare K-wires with plate osteosynthesis in a randomized controlled trial. According to the results of the current study, an ascending, conventional ESIN method, while conceptually appealing, cannot be recommended in the treatment of distal diametaphyseal radius fractures. The ESIN technique may be improved using a modified ESIN osteosynthesis with double buckling or using the descending technique.

## Figures and Tables

**Table 1:** Anthropometry and type of osteosynthesis.

	Gender [Male/Female]	Age [Year]	Body Size [Meter]	Weight [Kilogram]
K-wire	42/12	8.8 ± 3.2	1.47 ± 0.17	34.7 ± 12.6
Plate	89/28	10.6 ± 3.0	1.51 ± 0.18	46.5 ± 17.1
ESIN	28/13	8.2 ± 3.2	1.35 ± 0.19	33.0 ± 17.6
	*p* = 0.53	*p* < 0.0001	*p* < 0.01	*p* < 0.0001

**Table 2:** Description of the complications.

		Type of Osteosynthesis		
		K-Wire	Plate	ESIN
Clavien 1	Hypertrophic scar	0	20	2
	Periosteosynthetic infection	0	0	1
Clavien 2	Movement restriction for 3 to 6 months	1	0	2
	Material retention	1	0	0
	Neurology after surgery	1	0	0
	Periosteosynthetic infection (antibiotics)	2	0	0
Clavien 3	Movement restriction over 6 months	1	0	1
	Angular deformity radius with revision	0	0	2
	Iatrogenic tendon injury	1	0	1
	Peri-implant fracture	0	1	0
	Implant-associated fracture	0	1	0
	Refracture	0	3	0
	Migration of the osteosynthesis material	2	0	2
	Iatrogenic, intraoperative fracture	0	0	1
	Intraoperative change in procedure	0	0	1

**Table 3:** Classification of complications.

	K-Wire	ESIN	Plate
Slight complication = Clavien 1 and 2	5/54 (9%)	5/41 (12%)	20/118 (17%)
Serious complication = Clavien 3	4/54 (7%)	8/41 (20%)	5/118 (4%)

**Table 4:** Reduction results depending on the osteosynthesis procedure.

Reposition Result	Good	Moderate	Bad
K-wire	31 (57%)	17 (32%)	6 (11%)
Plate	110 (93%)	8 (7%)	0
ESIN	25 (61%)	14 (34%)	2 (5%)

## Data Availability

Research data supporting reported details is not online due to data protection restrictions. Research data can only be requested from the authors.

## Abbreviations

The following abbreviations are used in this manuscript:

ESIN	Elastic stable intramedullary nail
TEN	Titanium elastic nail
vs.	Versus
Clavien	Modified Clavien–Dindo–Sink classification
